# Database comments on Telegram channels related to cryptocurrencies with sentiments

**DOI:** 10.1186/s13104-024-06778-9

**Published:** 2024-05-14

**Authors:** Kia Jahanbin, Mohammad Ali Zare Chahooki, Mahdi Yazdian-Dehkordi, Fereshte Rahmanian

**Affiliations:** 1 Department of Computer Engineering, Yazd University, Yazd, Iran; 2 Department of Information Technology, Islamic Azad University Branch of Kerman, Kerman, Iran

**Keywords:** Telegram, Cryptocurrencies, Sentiment analysis, Trend prediction

## Abstract

**Objectives:**

Due to the limitations of Twitter, the expansion of Telegram channels, and the Telegram API’s easy use, Telegram comments have become prevalent. Telegram is one of the most popular social networks, unlike Twitter, which has no restrictions on sending messages, and experts can share their opinions and media. Some of these channels, managed by influencers of large companies, are very influential in the behavior of the market on various stocks, including cryptocurrencies. In this research, the opinion collection of 10 famous Telegram channels regarding the analysis of cryptocurrencies has been extracted. The sentiments of these opinions have been analyzed using the HDRB model. HDRB is a hybrid model of RoBERTa deep neural network, BiGRU, and attention layer used for sentiment analysis (SA). Analyzing the sentiments of these opinions is very important for understanding the future behavior of the market and managing the stock portfolio. The opinions of this dataset, published by experts in the field of cryptocurrencies, are precious, unlike the opinions that are extracted only by using the hashtag of the names of cryptocurrencies. On the other hand, the dataset related to cryptocurrencies, which has the opinions of experts and the polarity of their feelings, is very rare.

**Data description:**

The dataset of this research is the sentiments of more than ten popular Telegram channels regarding a wide range of cryptocurrencies. These comments were collected through the Telegram API from December 2023 to March 2024. This data set contains an Excel file containing the text of the comments, the date of comment creation, the number of views, the compound score, the sentiment score, and the type of sentiment polarity. These opinions cover influencer analysis on a wide range of cryptocurrencies. Also, two Word files, one containing the description of the dataset columns and the other Python code for extracting comments from Telegram channels, are included in this dataset.

**Supplementary Information:**

The online version contains supplementary material available at 10.1186/s13104-024-06778-9.

## Objective

Behavioral economics has proven that analyzing market behavior effectively predicts stock price trends [1]. It has also been shown in sate-of-arts [2–4] that sentiment analysis of comments on social networks such as X (former Twitter), Telegram, Reddit, and Facebook can effectively help predict the price trend of cryptocurrencies. In this article, Telegram comments of more than ten popular cryptocurrency-related channels have been extracted using the Telegram API from December 2023 to March 2024. Unlike the previous dataset [5–7] that are extracted only through the hashtag of the names of cryptocurrencies, this dataset contains the analysis of experts in cryptocurrencies, which is very effective on investors’ decisions in buying, selling, or holding cryptocurrencies. Unlike most existing datasets, this dataset covers a wide range of ciphers. Also, in addition to the main text of Telegram comments, this data set includes the number of views of each comment, the date of publication, and the polarity and the polarity score of the comments. After the extraction and preprocessing of Telegram comments, the polarity of these comments is determined by the HRDB model [8]. This model uses the RoBERTa pre-trained neural network as the backbone for transfer learning. Then, the extracted knowledge is injected into the deep neural network of BiGRU by combining the attention layer to determine the polarity of emotions. The main goal of this research is to provide a dataset of Telegram comments for the sentiment analysis of passwords, which has many applications in the training of neural networks for research in the field of passwords. This dataset helps researchers analyze the opinions of Telegram channels on a wide range of cryptocurrencies. The introduced data package includes an Excel table containing the Telegram monitoring set and two Word files. The Word files contain the descriptions of the columns of the main Table and Python code to extract comments from Telegram channels.

## Data description

This data package has three files. An Excel file contains the opinions of over ten popular Telegram channels about cryptocurrencies. The monitoring of these Telegram channels covers a wide range of cryptocurrencies from December 2023 to March 2024. It was collected through the Telegram API, and the code for extracting these comments is available in the Word package file. After extracting the comments, the operations were performed on them, including equalization, removing stop words, and lemmatization. Then, these data are injected into the HDRB model, described in detail in the research of Kia et al. [8], along with its implementation method. HDRB is a hybrid model based on transfer deep learning that uses the RoBERTa as a backbone and feature extractor and BiGRU deep neural network and attention layer to obtain sentiment polarity and text aspects. This dataset package and Python codes for pre-processing and extracting Telegram comments are listed in Table (1).

**Table 1:** Overview of data files/data sets

Label	Name of data file/data set	File types (file extension)	Data repository and identifier (DOI or accession number)
*Data set 1*	*telegram_channels_messages14021213_with_sentiment*	*Excel (.csv)*	Mendeley Data (10.17632/3733zt5bs6.1) [9]
*Meta Data*	*Column_Description*	*Word (.docx)*	Mendeley Data (10.17632/3733zt5bs6.1) [9]
*Python code*	*Extraction of Telegram’s comments and Preprocessing*	*Word (.docx)*	Mendeley Data (10.17632/3733zt5bs6.1) [9]

The information of Dataset 1 is (1) text, (2) date, (3) views, (4) scores, (5) compound, and (6) sentiment_type. In the mentioned features, “text” is the preprocessed Telegram comment, “date” column shows the time and date of publication of the comment, “views” shows the number of people’s views of a comment, " scores” shows the percentage of positive, negative, and neutral polarities. These percentages were obtained with the HDRB model [8], “compound” shows the sum of all polarities in a normalized form between − 1 (most extreme negative) and + 1 (most extreme positive), and " sentiment_type” It shows the type of tweet polarity (positive, negative, or neutral). Researchers can easily change the number of polarities by using compound values—for example, strongly positive, positive, neutral, negative, and strongly negative.

## Limitations

There are no limitations in the datasets, and the Telegram channels used in the datasets to extract Telegram’s comments are public.

### Electronic supplementary material

Below is the link to the electronic supplementary material.

Supplementary Material 1

## Data Availability

You can access the data package, including the dataset and other required files, for free through the link below. Link: https://data.mendeley.com/datasets/3733zt5bs6/1.

## References

[1] Thaler R. *Behavioral economics: Past, present, and future* 2016. 106(7): pp. 1577–1600.

[2] Parekh R et al. *DL-GuesS: Deep learning and sentiment analysis-based cryptocurrency price prediction* 2022. 10: pp. 35398–35409.

[3] Valencia F, Gómez-Espinosa A, Valdés-Aguirre B (2019). Price Mov Prediction Cryptocurrencies Using Sentiment Anal Mach Learn.

[4] Wołk K. Advanced social media sentiment analysis for short-term cryptocurrency price prediction. 2020. 37(2): p. e12493.

[5] KANNIAH G. *Pre-processed Tweets by verified users, Elon Musk, Vitalik Buterin and CZ Binance*. 2021, 10.5281/zenodo.5336611.

[6] Mazzoli I. 2022: Kaggle, https://www.kaggle.com/datasets/ilariamazzoli/3-million-tweets-cryptocurrencies-btc-eth-bnb.

[7] Peleg Y. Cryptocurrency extra data-Elon musk’s tweets. Kaggle; 2021. https://www.kaggle.com/yamqwe/elon-musks-twitter-updated-031121.

[8] Jahanbin K, Chahooki MAZ. *Aspect-Based Sentiment Analysis of Twitter Influencers to Predict the Trend of Cryptocurrencies Based on Hybrid Deep Transfer Learning Models* 2023. 11: pp. 121656–121670.

[9] Jahanbin K, Chahooki MAZ, Rahmanian F, Yazdian M, Dehkordi. Comments on Telegram channels related to cryptocurrencies along with sentiments. data Mendeley. 2024;1. 10.17632/3733zt5bs6.1.

