# Supplementary material for: Database comments on Telegram channels related to cryptocurrencies with sentiments
Source: BMC Res Notes. 2024 May 14;17:135. doi: 10.1186/s13104-024-06778-9 (PMC11092043; doi:10.1186/s13104-024-06778-9)
Supplement: Supplementary file 1 — Supplementary Material 1 [file 13104_2024_6778_MOESM1_ESM.docx]

#Import Packages

import configparser

import json

import asyncio

from datetime import date, datetime, timezone

from telethon import TelegramClient

from telethon.errors import SessionPasswordNeededError

from telethon.tl.functions.messages import (GetHistoryRequest)

from telethon.tl.types import (

PeerChannel

)

import pandas as pd

# Your credentials

api_id = your_id_id (int)

api_hash = 'your_api_hash'

#Password=958221

# Creating the client

client = TelegramClient('your_bot_name', api_id, api_hash)

async def extract_channel_messages(channel_name, start_time):

# Getting the channel entity

channel = await client.get_entity(channel_name)

# List to hold all messages

messages_data = []

# Getting the messages

async for message in client.iter_messages(channel):

# Check if the message is later than the start time

if message.date > start_time.replace(tzinfo=timezone.utc): # Make start_time offset-aware

messages_data.append({

'channel': channel_name,

'id': message.id,

'text': message.text,

'date': message.date,

'views': message.views

})

return messages_data

async def main(start_time_str):

# Connecting and logging in

await client.start(phone='your_phone_number', password='your_password')

# User-specified start time

start_time = datetime.strptime(start_time_str, '%Y-%m-%d').replace(tzinfo=timezone.utc) # Make start_time offset-aware

# Channels to extract from

channels = ['Channel_1', 'channel_2','Channel_n']

all_messages = []

for channel in channels:

messages = await extract_channel_messages(channel, start_time)

all_messages.extend(messages)

# Creating a DataFrame

df = pd.DataFrame(all_messages)

# Save the DataFrame to a CSV file with channel name

df.to_csv('telegram_channels_messages.csv', index=False, encoding='utf-8')

# Logout and disconnect

await client.log_out()

await client.disconnect()

# User input for start time

user_start_time = input("Enter start time (YYYY-MM-DD): ")

# Running the client

async with client:

await main(user_start_time)
